# Resting-state functional connectivity and pitch identification ability in non-musicians

**DOI:** 10.3389/fnins.2015.00007

**Published:** 2015-02-11

**Authors:** Jiancheng Hou, Chuansheng Chen, Qi Dong

**Affiliations:** ^1^State Key Lab of Cognitive Neuroscience and Learning, Beijing Normal UniversityBeijing, China; ^2^Department of Psychology, Ohio State UniversityColumbus, OH, USA; ^3^Department of Psychology and Social Behavior, University of California, IrvineIrvine, CA, USA

**Keywords:** resting-state functional connectivity, pitch identification, musical training, non-musicians

## Abstract

Previous studies have used task-related fMRI to investigate the neural basis of pitch identification (PI), but no study has examined the associations between resting-state functional connectivity (RSFC) and PI ability. Using a large sample of Chinese non-musicians (*N* = 320, with 56 having prior musical training), the current study examined the associations among musical training, PI ability, and RSFC. Results showed that musical training was associated with increased RSFC within the networks for multiple cognitive functions (such as vision, phonology, semantics, auditory encoding, and executive functions). PI ability was associated with RSFC with regions for perceptual and auditory encoding for participants with musical training, and with RSFC with regions for short-term memory, semantics, and phonology for participants without musical training.

## Introduction

Some professional musicians can identify a single musical note quickly and accurately without the benefit of a reference note. They are considered as having absolute pitch (AP) (Levitin, 1999; Parncutt and Levitin, 2001). Neuroimaging studies have shown that AP musicians have a reduced P300 (Nishitani et al., 1998; Hirose et al., 2002), an increased cerebral blood flow (CBF) in the left posterior dorsolateral frontal cortex (Zatorre et al., 1998), and enhanced white matter connectivity and increased fractional anisotropy in the left superior longitudinal fasciculus (Oechslin et al., 2010).

In addition to AP musicians, quasi-AP musicians (i.e., those with a weak form of AP) have also been studied. Using the PET, Wilson et al. (2006, 2009) found that, during a pitch identification (PI) test, quasi-AP musicians had significant activations within an extensive right hemisphere network, including the right superior and middle temporal gyri, right dorsolateral prefrontal cortex, right middle and inferior frontal gyri, and right cerebellum. In contrast to the left hemispheric advantage in AP musicians as mentioned in the previous paragraph, Wilson et al. (2009) showed that the right hemisphere was important for PI in quasi-AP musicians.

Thus, far, little is known about the neural basis of PI among non-musicians. Only one study by Schwenzer and Mathiak (2011) collected fMRI data while non-musicians were asked to recognize a single pitch from a set of four frequencies in each trial. The results showed that PI activated the right dorsolateral prefrontal cortex (DLPFC), right medial frontal gyrus, right medial front lobe, bilateral premotor area, and bilateral intraparietal sulcus (IPS). These regions subserve various functions involved in PI: the DLPFC for working memory (Crottaz-Herbette et al., 2004; Grimault et al., 2009); the medial frontal gyrus for error monitoring (Volz et al., 2005); the medial frontal lobe for retaining memory, executive function and attention (Simons and Spiers, 2003; Baird et al., 2006); the premotor area for planning movement (Churchland et al., 2006; Ojakangas et al., 2006); and the IPS for spatial and quantity processing (Castelli et al., 2006; Dormal and Pesenti, 2009; Santens et al., 2010; Schwenzer and Mathiak, 2011). As was the case for quasi-AP musicians, the right hemisphere was important for PI among non-musicians.

Thus, far, no study has examined the role of resting-state functional connectivity (RSFC) among different brain regions in either AP or PI. Resting-state fMRI measures the low-frequency (~0.01–0.1 Hz) spontaneous neuronal activity in the brain (Lv et al., 2008) and is believed to reflect neuronal function (Damoiseaux et al., 2006; Fox and Raichle, 2007; Zhang et al., 2014). Thus, far, the only RSFC studies in musicological research were those that aimed to characterize the motor systems in musicians. For example, Lv et al. (2008) found significant RSFC between the left and right primary sensorimotor areas in pianists. Luo et al. (2012) found significant RSFC between the motor and multi-sensory cortices (such as visual, auditory, and somatosensory cortices) in musicians, which may reflect their enhanced functional integration among the lower-level perceptual and motor networks as well as the functional consolidation (plasticity) due to long-term music training.

In the current study, we examined the RSFC differences between the non-musicians with and without musical training and the correlations between PI ability and RSFC in a large sample of Chinese non-musicians.

## Methods and materials

### Participants

Data for the current study came from a larger project conducted with students from Beijing Normal University. 320 undergraduate students (age range: 19–24 years, mean age = 20.45, *SD* = 1.18, 191 female and 129 male) had both RSFC data and data on a PI test. Among them, 56 participants (11 males and 45 females) had musical training experience (such as piano, keyboard, violin, accordion, etc.), and 264 participants (118 males and 146 females) had no musical training experience. Table 1 shows participants' basic demographic information.

**Table 1:** **Characteristics of the participants**.

**Characteristics**	**Mean (*SD*)**	**Range**
Age (years)	20.45 (1.18)	19–24
Gender (male/female)	320 (129/191)	
Handedness	All right-handed	
PI accuracy rate (%)	37.56 (5.13)	4.76–100
PI reaction time (millisecond)	1816.45 (423.97)	641–7187
Music training experience		
Musical training		
Starting age	10.67 (1.44)	8–20
Gender (male/female)	56 (11/45)	
PI accuracy rate (%)	58.80 (5.42)	9.52–100
PI reaction time (millisecond)	1704.11 (321.78)	738.14–4174.42
No musical training		
Gender (male/female)	264 (118/146)	
PI accuracy rate (%)	31.82 (9.49)	4.76–100
PI reaction time (millisecond)	1859.23 (435.64)	641–7187

*Note: Standard deviations are shown in parentheses*.

All participants had normal or corrected-to-normal vision and no history of neurological or psychiatric diseases. They were all right-handed as judged by Snyder and Harris's handedness inventory (Snyder and Harris, 1993). Informed written consent was obtained from all participants before scanning. This study was approved by the Institutional Review Board (IRB) of the State Key Laboratory of Cognitive Neuroscience and Learning at Beijing Normal University.

### Pitch identification test

**Figure 1:**
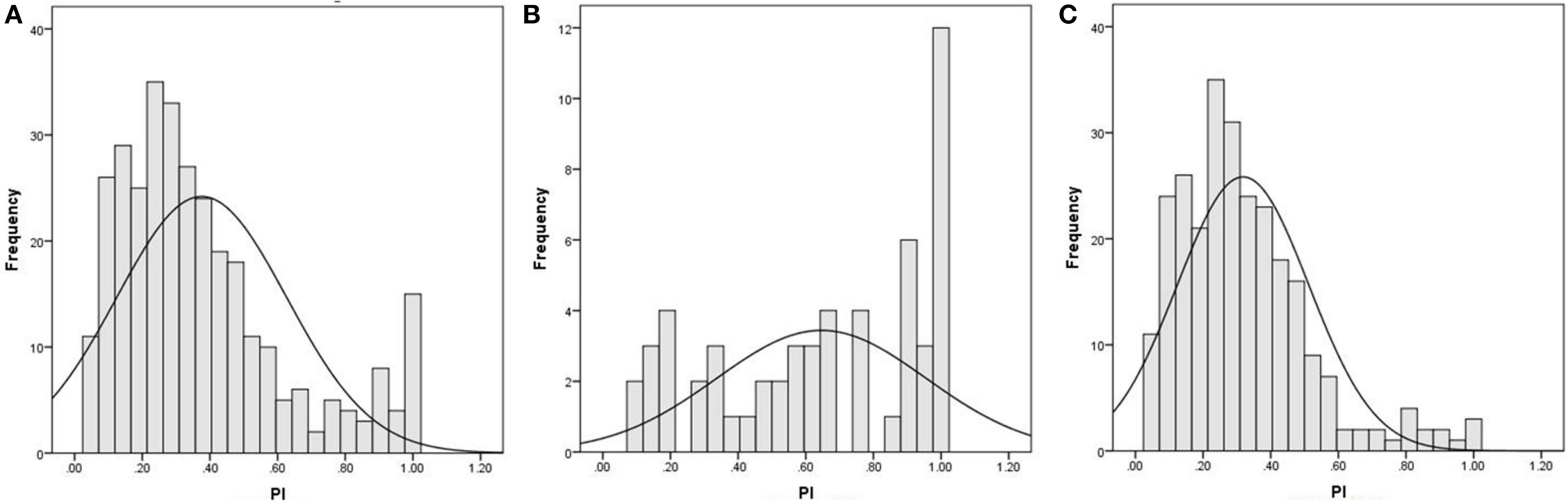
**The distribution of participants' accuracy rate on the pitch identification test. (A)** the total sample of 320 participants; **(B)** the participants with musical training; **(C)** the participants without musical training.

The PI test was adapted from the AP test developed by Zatorre (2003). Because the majority of the participants had no formal music training, only seven basic music notes from the fifth octave were used (i.e., C4, D4, E4, F4, G4, A4, and B4, with corresponding frequencies of 261.60, 293.66, 329.63, 349.23, 392.00, 440.00, and 494.88 Hz, respectively). Participants responded by clicking a corresponding key on the computer screen after listening to a note. The seven pitches were randomly presented. Each pitch was presented for 500 ms twice and was tested three times. The inter-stimulus interval was 1000 ms. Before the formal test, there was a practice test for 5 min and participants were given feedback (i.e., they were told which note had been presented), again because most of the participants had no music training. The formal test took about 10 min to complete and it was conducted without feedback. Accuracy rate and reaction time during the formal test were collected (also see Hou et al., 2014). In current study we analyzed the accuracy rate (see Table 1 for mean PI scores, standard deviations, and range; see Figure 1 for distributions of the PI scores by group).

### MRI data acquisition

Data were acquired with a 3.0 T Siemens MRI scanner in the MRI Center of Beijing Normal University. A single-shot T2*-weighted gradient echo EPI sequence was used for a brief scan (8 min) which comprised 240 continuous echo planar imaging functional volumes with the following parameters: TR/TE/θ = 2000 ms/25 ms/90°, FOV = 192 × 192 mm, matrix = 64 × 64, and slice thickness = 3 mm. During the scan, participants laid supine on the scanner bed. Foam pads were used to minimize head motion. Participants were instructed to close their eyes, keep their head still, think about nothing in particular, and just relax. We determined whether participants were awake during scanning by talking to the participants immediately after the session. If they responded immediately and reported that they stayed awake during the scan, we assumed they were awake. Of all the participants in the original larger study, one participant was determined to have slept during scanning, whose data were not included in the database. Anatomical MRI was acquired using a T1-weighted, three-dimensional, gradient-echo pulse-sequence (MPRAGE) with TR/TE/θ = 2530 ms/ 3.09 ms/10°, FOV = 256 × 256 mm, matrix = 256 × 256, and slice thickness = 1 mm. Two hundred and eight sagittal slices were acquired to provide high-resolution structural images of the whole brain.

### Region of interest (ROI) selection

**Figure 2:**
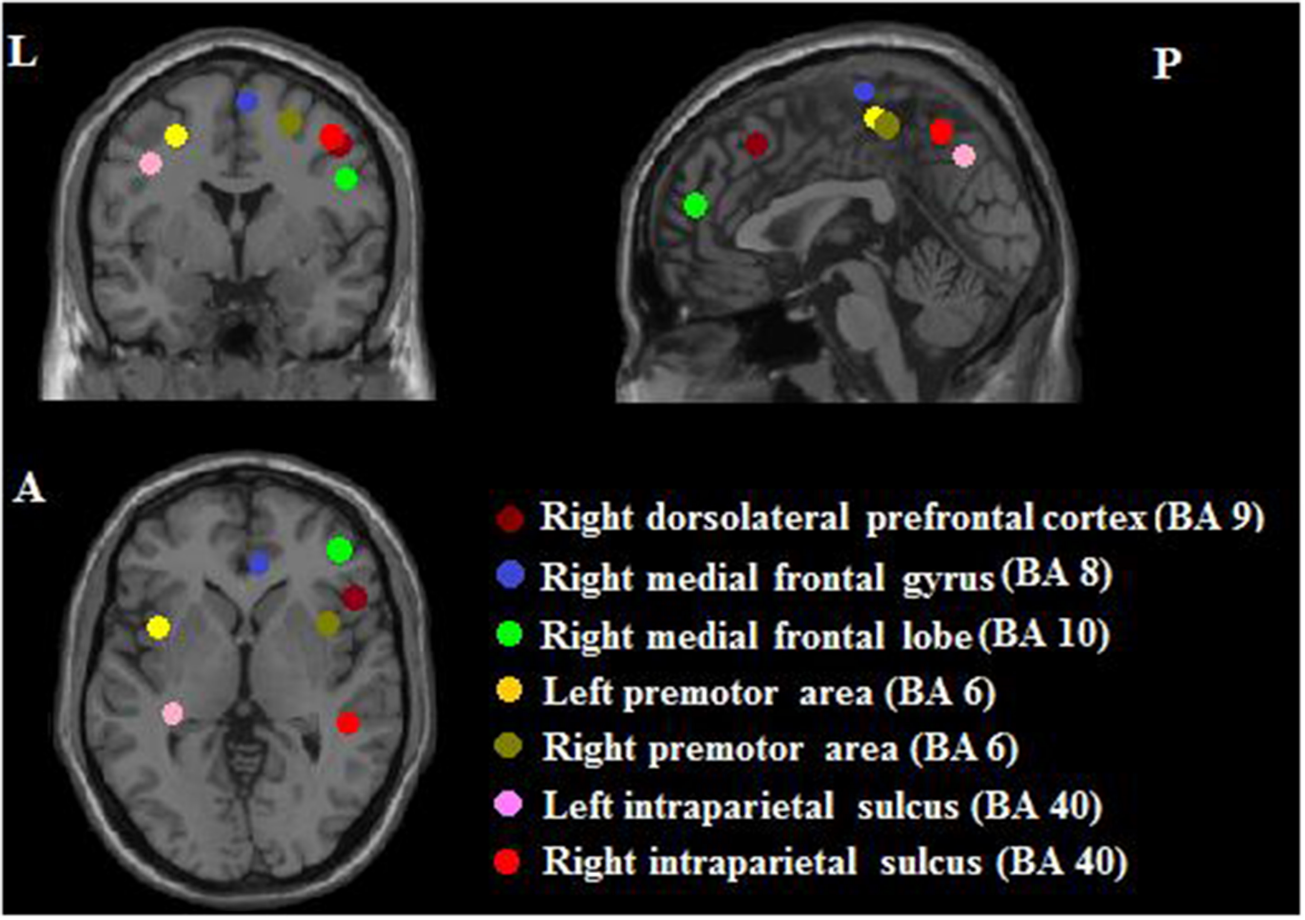
**Seven seed ROIs. L, left; A, anterior; P, posterior**.

Because the current study did not collect task-related fMRI, we selected seed regions based on a previous task-related fMRI study for PI among non-musicians (Schwenzer and Mathiak, 2011). That study found seven significant ROIs (with MNI coordinates indicated): right dorsolateral prefrontal cortex (*x* = 40, *y* = 20, *z* = 36), right medial frontal gyrus (*x* = 4, *y* = 18, *z* = 50), right medial frontal lobe (*x* = 34, *y* = 48, *z* = 8), left premotor area (*x* = −30, *y* = −6, *z* = 50), right premotor area (*x* = 24, *y* = −2, *z* = 52), left intraparietal sulcus (*x* = −36, *y* = −34, *z* = 40), and right intraparietal sulcus (*x* = 40, *y* = −40, *z* = 44). The radius was 6 mm. Please see all ROIs in Figure 2.

### Data preprocessing

Image preprocessing was carried out using Data Processing Assistant for Resting-State fMRI (DPARSF) (http://www.nitrc.org/projects/dparsf/) version 2.2. DPARSF is a convenient plug-in software based on Statistical Parametric Mapping (SPM) and Resting-State fMRI Data Analysis Toolkit (REST) (http://www.restfmri.net). The Digital Imaging and Communications in Medicine (DICOM) files were first arranged and the parameters (such as time points, TR, slice number, voxel size et al.) were then set. DPARSF then produced the preprocessed data (with slice timing, realignment, normalization, and smoothing) and the results of functional connectivity (FC), regional homogeneity (ReHo), amplitude of low-frequency fluctuation (ALFF) and fractional ALFF (fALFF). The current study used the FC analysis. The first 10 volumes were discarded to allow the magnetisation to approach a dynamic equilibrium, and for the participants to get used to the scanner noise. No participants showed head motion above 3.0 mm of maximal translation (in any direction of *x, y*, or *z*) and 2.5° of maximal rotation throughout the course of scanning (Yan et al., 2009). Data pre-processing, including slice timing, realignment, normalization, smoothing, regressing out head motion parameters (using a least squares approach and a 6-parameter spatial transformation), and spatial normalization to the Montreal Neurological Institute (MNI) template (resampling voxel size of 3 × 3 × 3 mm), were conducted using SPM8 and DPARSF version 2.2 (Yan and Zang, 2010; Kuhn et al., 2012). A spatial filter of 5 mm FWHM (full-width at half maximum) was used.

### Statistical analysis

SPSS 16.0 version was used to analyze the behavioral data. For the RSFC analysis, the Resting-State fMRI Data Analysis Toolkit (REST) (http://www.restfmri.net) was used (Yan and Zang, 2010; Song et al., 2011). To examine the music training effects, we conducted independent-sample *t*-tests on a whole brain *Z*-value map between the participants with and without musical training. Within each group, we then correlated the *Z*-value map with the PI score. Gender was included as a covariate. Monte Carlo simulations were performed using the AFNI AlphaSim program for multiple comparison correction. By iterating the process of random image generation, spatial correlation of voxels, thresholding, and cluster identification, the program provides an estimate of the overall significance level achieved for various combinations of individual voxel probability threshold and cluster size threshold (Bennett et al., 2009; Wu et al., 2011). Using this program, a threshold correction adjustment was used with a voxel-wise *p* < 0.05, 1000 simulations, cluster size > 212 (5724 mm^3^). Because the interpretations of negative RSFC (or anti-correlations) are still being debated and their neuronal basis is unclear (e.g., Weissenbacher et al., 2009; but for recent developments, see Chai et al., 2012; Liang et al., 2012), we focused on our analyses on positive RSFC. In addition, we focused on positive behavioral correlates of these RSFC and presented the negative behavioral correlates in the Supplemental Online Materials.

## Results

The mean accuracy rate on the PI test was 37.56% (*SD* = 5.13), ranging from 4.76 to 100% (15 participants had 100%) (see Figure 1A). For the participants with musical training, the mean accuracy rate on the PI test was 65.19% (*SD* = 3.09), ranging from 9.52 to 100% (12 participants had 100%, Figure 1B). For the participants without musical training, the mean was 31.82% (*SD* = 9.49), ranging from 4.76 to 100% (3 participants had 100%, Figure 1C). The group difference was significant, *t*_(318)_ = 10.21, *p* < 0.001.

**Figure 3:**
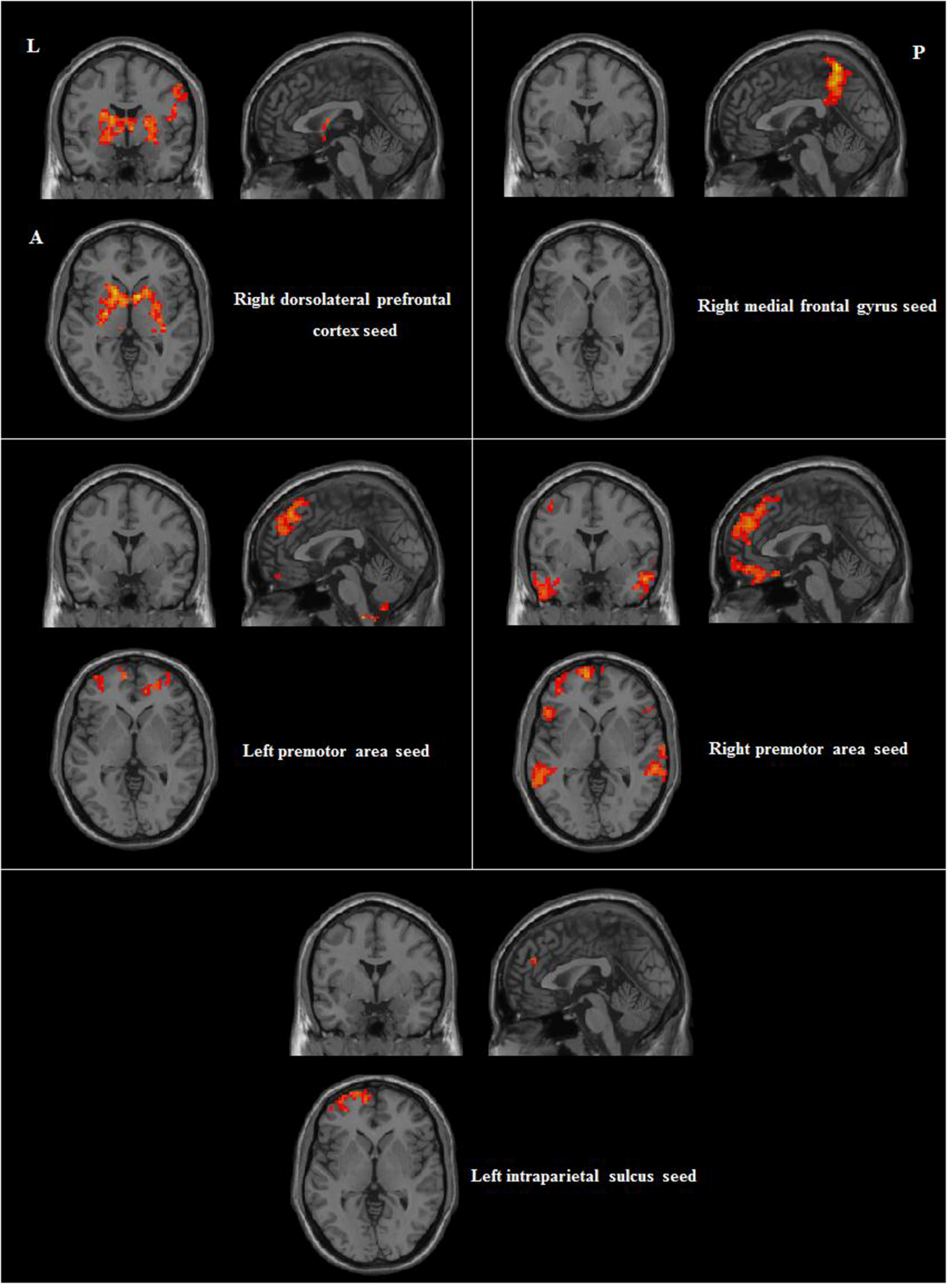
**Differences in positive RSFC between participants with and without musical training (AlphaSim corrected *p* < 0.05, cluster size > 212)**. L, left; A, anterior; P, posterior.

We then compared group differences in RSFC between the seed regions and other brain areas. Compared to participants without musical training, those with musical training showed stronger RSFC between the right dorsolateral prefrontal cortex seed and the bilateral superior temporal gyri and right inferior parietal lobule; between the right medial frontal gyrus seed and the right precuneus; between the left premotor area seed and the right cerebellum, right superior medial frontal gyrus, and left pars triangularis; between the right premotor area seed and the bilateral inferior temporal gyrus, right pars triangularis, right cerebellum, and left middle frontal gyrus; and between the left intraparietal sulcus seed and the left middle frontal gyrus. Detailed information of these results are shown in Table 2 and Figure 3.

**Table 2:** **Positive differences in RSFC between participants with and without musical training**.

**ROI seeds**	**Cluster location**	**BA**	**Peak (MNI)**			**Cluster size**	***t***
			***x***	***y***	***z***		
Right dorsolateral prefrontal cortex	Right superior temporal gyrus	48	42	−27	66	252	4.15
	Right inferior parietal lobule	40	54	−45	39	257	3.23
	Left superior temporal gyrus	48	−18	14	−6	246	3.35
Right medial frontal gyrus	Right precuneus	5	0	−48	63	457	4.39
Left premotor area	Right cerebellum		6	−60	−45	224	4.49
	Right superior medial frontal gyrus	8	9	30	60	400	3.73
	Left pars triangularis	45	−33	42	−6	213	4.07
Right premotor area	Right inferior temporal gyrus	20	63	−18	−21	616	4.40
	Right pars triangularis	45	45	45	−15	320	4.33
	Right cerebellum		42	−66	−45	241	4.09
	Left inferior temporal gyrus	20	−60	−18	−24	2005	4.79
	Left middle frontal gyrus	9	−39	18	48	1429	5.25
Left intraparietal sulcus	Left middle frontal gyrus	46	−33	18	45	380	4.29

*Note: AlphaSim corrected p < 0.05, cluster size > 212*.

**Figure 4:**
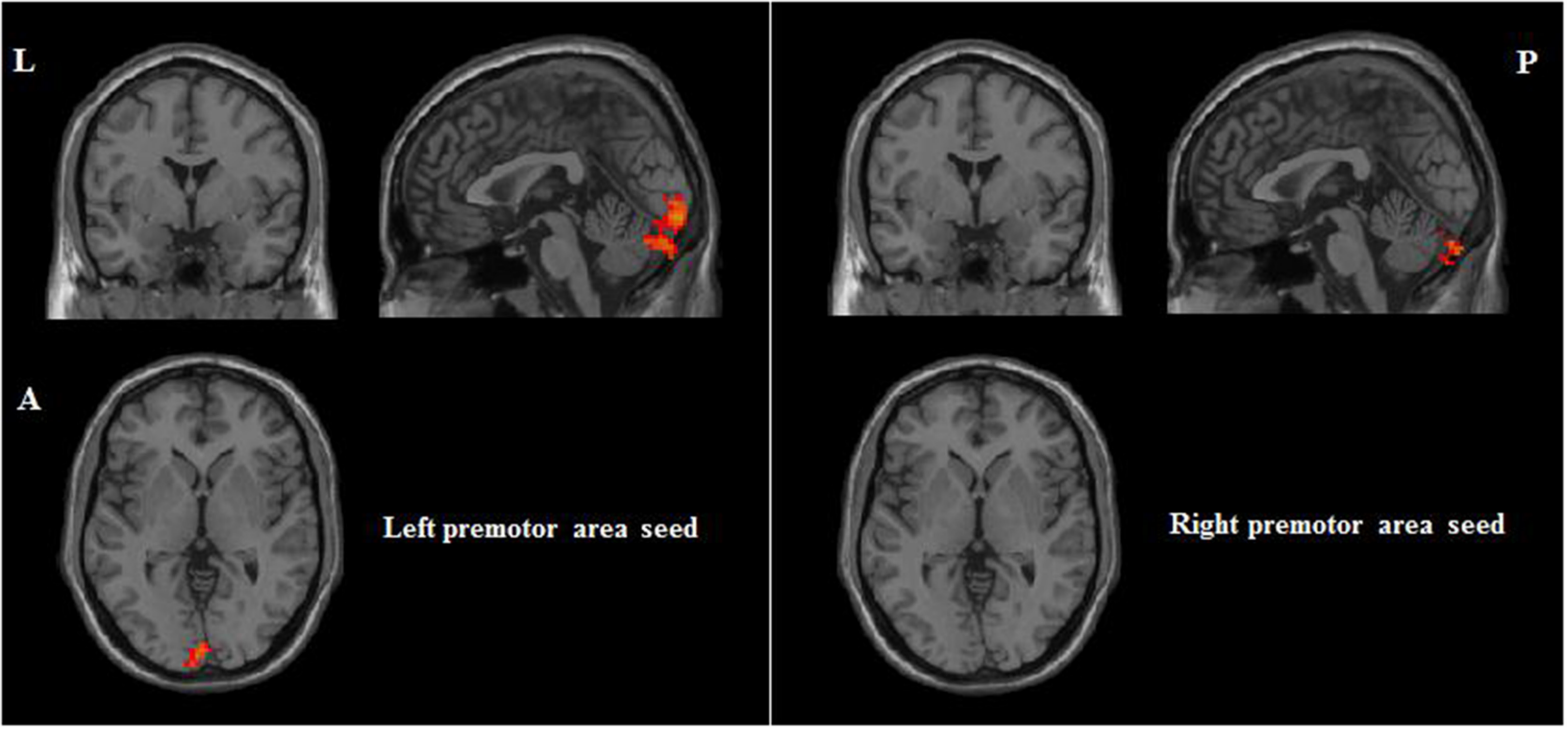
**Positive correlations between RSFC and PI for participants with musical training (AlphaSim corrected *p* < 0.05, cluster size > 212)**. L, left; A, anterior; P, posterior.

**Figure 5:**
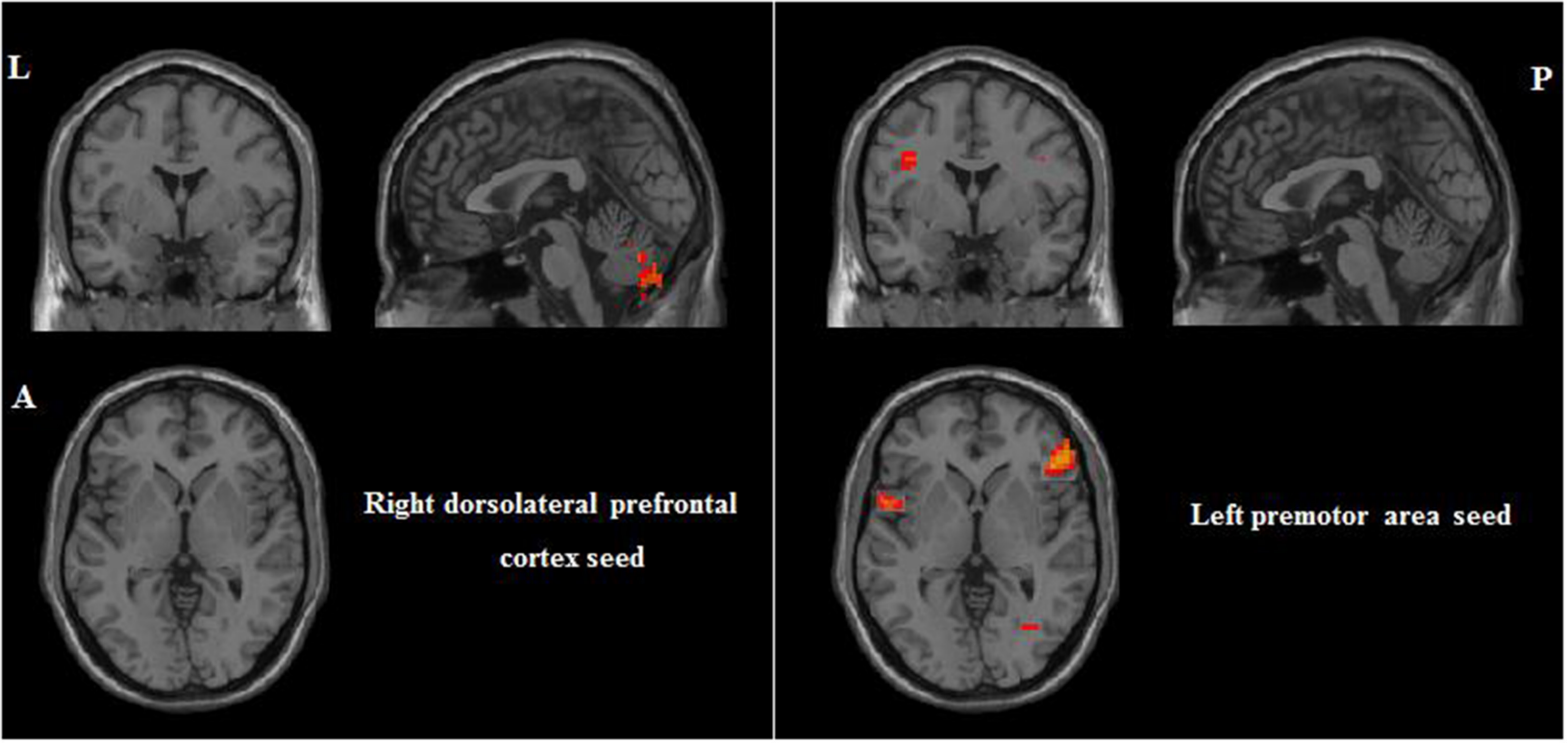
**Positive correlations between RSFC and PI for participants without musical training group (AlphaSim corrected *p* < 0.05, cluster size > 212)**. L, left; A, anterior; P, posterior.

Correlations between RSFC and PI were obtained for each group of participants. For participants with musical training, positive correlates of PI ability included RSFC between the bilateral premotor area seeds and the left cerebellum (see Table 3 and Figure 4). For participants without musical training, positive correlates of PI ability included RSFC between the right dorsolateral prefrontal cortex seed and the right cerebellum, and between the left premotor area seed and the bilateral inferior parietal lobule, right pars triangularis, and left superior temporal gyrus (see Table 3 and Figure 5).

**Table 3:** **Positive correlations between RSFC and PI for the two groups**.

**ROI seeds**	**Cluster location**	**BA**	**Peak (MNI)**			**Cluster size**	***r***
			***x***	***y***	***z***		
**PARTICIPANTS WITH MUSICAL TRAINING**							
Left premotor area	Left cerebellum		−33	−87	−27	725	0.58
Right premotor area	Left cerebellum		−30	−87	−27	317	0.46
**PARTICIPANTS WITHOUT MUSICAL TRAINING**							
Right dorsolateral prefrontal cortex	Right cerebellum		9	−78	−45	298	0.24
Left premotor area	Right pars triangularis	45	51	33	24	271	0.23
	Right inferior parietal lobule	40	33	−48	42	251	0.22
	Left superior temporal gyrus	48	−48	21	27	227	0.23
	Left inferior parietal lobule	7	−30	−66	54	323	0.28

*Note: AlphaSim corrected p < 0.05, cluster size > 212*.

In addition to the positive relationships between RSFC and cognitive performance, our analysis also revealed several significant negative relationships between RSFC and musical training and between RSFC and PI ability. These results are presented in Supplementary Tables S1, S2 and Supplementary Figures S1–S3. Finally, because our ROIs were selected based on a previous task fMRI study, which might have missed important seed regions, we conducted a whole-brain analysis across the Automated Anatomical Labeling (AAL) ROIs. Additional associations between RSFC and musical training and between RSFC and PI ability were identified. These results and a brief discussion are presented in Supplementary Tables S3, S4.

## Discussion

Using a large sample of non-musicians, the current study aimed to examine how RSFC was associated with musical training and PI ability.

### Music training and RSFC

Whole-brain analysis revealed significant RSFC differences between participants with and without musical training. First, musical training was associated with increased functional connectivity linking the right DLPFC seed to the bilateral superior temporal gyrus and right inferior parietal lobule (the latter is within the default mode network). These results are aligned with previous findings of music training effects on these regions' functions in language processing. For example, musical training has been found to improve phonological and semantic processing in the superior temporal gyrus (Platel et al., 2003; Lai et al., 2014), phonological processing in the inferior parietal lobule (Bermudez and Zatorre, 2005; Bermudez et al., 2009; Lai et al., 2014), and executive functions in the inferior parietal lobule (Bermudez and Zatorre, 2005).

Second, musical training was associated with increased functional connectivity linking the right medial frontal gyrus seed to the right precuneus. The precuneus, another region within the default mode network, is responsible for musical memory, imagery and emotion (Demorest et al., 2010). Previous research has shown that musical training can improve this region's visual “mental imagery” in a pitch change task (Platel et al., 1999; Meister et al., 2004).

Third, the participants with musical training showed significantly increased functional connectivity linking the bilateral premotor area seeds to the regions that subserve the functions of object recognition (bilateral inferior temporal gyrus, Bogousslavsky et al., 1987; Heywood et al., 1995; Olson et al., 2007), auditory processing and speech (bilateral inferior temporal gyrus, bilateral pars triangularis, McGuire et al., 1995; Onitsuka et al., 2004; Kaplan et al., 2010; Romanski, 2012), the maintenance and selective retrieval of memory components (left middle frontal gyrus, Bermudez and Zatorre, 2005), executive functions (right superior medial frontal cortex, Talati and Hirsch, 2005), and behavior or movement control and auditory encoding (right cerebellum, Gaab et al., 2003; Petacchi et al., 2005; Schulze et al., 2009). This cluster of strengthened RSFC may reflect the benefits of music training on cognitive abilities such as vision, kinesthesia, motion perception, verbal processing, and even higher cognitive functions (Zatorre and Beckett, 1989; Zhou, 2004; Bermudez and Zatorre, 2005).

Finally, the participants with musical training showed significantly increased functional connectivity linking the left intraparietal sulcus seed to the left middle frontal gyrus. The IPS is mainly responsible for musical, spatial, and quantity processing (Cappelletti et al., 2007; Husain and Nachev, 2007; Offen et al., 2010; Cheng et al., 2013). Foster and Zatorre (2010) found an increased activation in the intraparietal sulcus for music note processing, perhaps involving the visual-spatial mapping scheme (i.e., imagining notes on a staff or using a spatial coding for their relative pitch height) during pitch processing (also see Zhou, 2004; Rusconi et al., 2005; Williamson et al., 2011). Schulze et al. (2009) found that the middle frontal gyrus was involved in the tonal working memory. It seems that music training strengthened connectivities between the above two regions and consequently pitch perception and memory (Zatorre and Beckett, 1989; Gaab et al., 2003; Zhou, 2004).

### RSFC and PI ability

For participants without musical training, there were two main findings. First, there was a positive correlation between the right dorsolateral prefrontal cortex seed and the right cerebellum. The cerebellum, traditionally viewed as a motor structure, is found to be active in a wide variety of sensory and cognitive tasks. Schulze et al. (2009) found that a music pitch memory task elicited cerebellar activations in both AP and non-AP musicians (also see Gaab et al., 2003). It has also been found to be involved in higher cognitive processes such as working memory (Baddeley, 2003; Marvel et al., 2012) and multimodal encoding (Stewart et al., 2003; Cullen, 2012; Billings et al., 2014). In a meta-analysis of 15 PET and fMRI auditory studies, Petacchi et al. (2005) found that a variety of auditory tasks consistently activated the cerebellum. The RSFC-PI relationship between the dorsolateral prefrontal cortex and the cerebellum perhaps reflects the role of this functional connectivity in pitch memory and discrimination (Gaab et al., 2003).

Second, there were significant correlations between PI ability and RSFC linking the left premotor area seed to the right pars triangularis, left superior temporal gyrus, and bilateral inferior parietal lobule. These three regions are responsible for language processing: the pars triangularis and superior temporal gyrus for semantic processing (Kaplan et al., 2010; Romanski, 2012; Lai et al., 2014), and the superior temporal gyrus and inferior parietal lobule for phonological processing (Hickok and Poeppel, 2004, 2007; Scott and Wise, 2004; Bermudez and Zatorre, 2005; Limb et al., 2006; Bermudez et al., 2009; Romanski, 2012). As mentioned earlier, these regions have been shown to be affected by music training. Interestingly, the RSFC between these regions and the DLPFC seed was associated with music training as reported in the previous section, but the RSFC between these regions and the premotor area seed was associated with PI ability for participants without music training. Further research is needed to explicate these differential associations.

For the participants with musical training, whose sample size was relatively small, there were only two significant associations: between the bilateral premotor area seeds and the left cerebellum. These functional connectivities suggest that better PI ability may rely on stronger connection between auditory encoding at the cerebellum to movement preparation and control at the premotor area (e.g., Gaab et al., 2003; Petacchi et al., 2005; Schulze et al., 2009).

### Limitations of the current study

Several limitations of the current study need to be noted. First, in order to accommodate the non-musician participants, we used a PI test that had a restricted range of notes and included practice trials. Thus, our results cannot be generalized to AP musicians. Second, because we did not include task-related fMRI for PI in this study, we relied on a previous study for ROI selection (as well as AAL for additional ROIs in the supplementary result), which might have missed important seed regions for our participants. Third, our sample size of the participants with music training was small and thus had less statistical power, which might have contributed to the divergent results between the two groups of participants. Fourth, our data were correlational, so it was not clear whether there were causal relations among musical training, PI ability, and RSFC. Fifth, because there was no effective way to monitor whether participants slept during the resting-state scanning other than immediate post-scan self-report, it was uncertain how many participants might have drifted away from wakefulness toward sleep, a concern that has been raised recently about RSFC studies (Tagliazucchi and Laufs, 2014). Finally, our participants were Chinese whose native language is a tonal language. Previous research has shown that speakers of tonal languages might have an advantage in AP or PI ability (Deutsch et al., 2004; Gandour et al., 1998; also see Bidelman et al., 2013, for an advantage of Cantonese speakers). Therefore, our results need to be replicated among speakers of non-tonal languages.

## Summary

With a large sample of Chinese non-musicians, the current study compared RSFC differences between participants with and without musical training and correlated RSFC with PI ability within each group. The results showed that musical training was associated with increased RSFC within the networks for multiple cognitive functions, such as vision, phonology, semantics, auditory encoding, and executive functions. Moreover, PI ability was associated with RSFC with regions for perceptual and auditory encoding for participants with musical training, and with RSFC with regions for short-term memory, semantics, and phonology for participants without musical training.

### Conflict of interest statement

The authors declare that the research was conducted in the absence of any commercial or financial relationships that could be construed as a potential conflict of interest.
