# Supplementary material for: Resting-state functional connectivity and pitch identification ability in non-musicians
Source: Front Neurosci. 2015 Feb 11;9:7. doi: 10.3389/fnins.2015.00007 (PMC4324073; doi:10.3389/fnins.2015.00007)
Supplement: Supplementary file 1 [file Presentation1.PDF]

Supplementary Material

Resting-state functional connectivity and pitch identification ability in non-musicians

Jiancheng Hou[1,2*], Chuansheng Chen[3*], Qi Dong[1]

[1] State Key Lab of Cognitive Neuroscience and Learning, Beijing Normal University, Beijing 100875, China

[2] Department of Psychology, Ohio State University, Columbus, Ohio 43224, United States

[3] Department of Psychology and Social Behavior, University of California, Irvine, California 92697, United States

* Jiancheng Hou and Chuansheng Chen contribute equal to this work.

Correspondence:

Chuansheng Chen

4566 Social and Behavioral Sciences Gateway, Department of Psychology and Social Behavior, University of California, Irvine, CA

92697, USA

e-mail: chuansheng.chen@uci.edu;

Qi Dong

Yingdong Building, State Key Lab of Cognitive Neuroscience and Learning, Beijing Normal University, Beijing 100875,

China

e-mail: dongqi@bnu.edu.cn

Supplementary Results

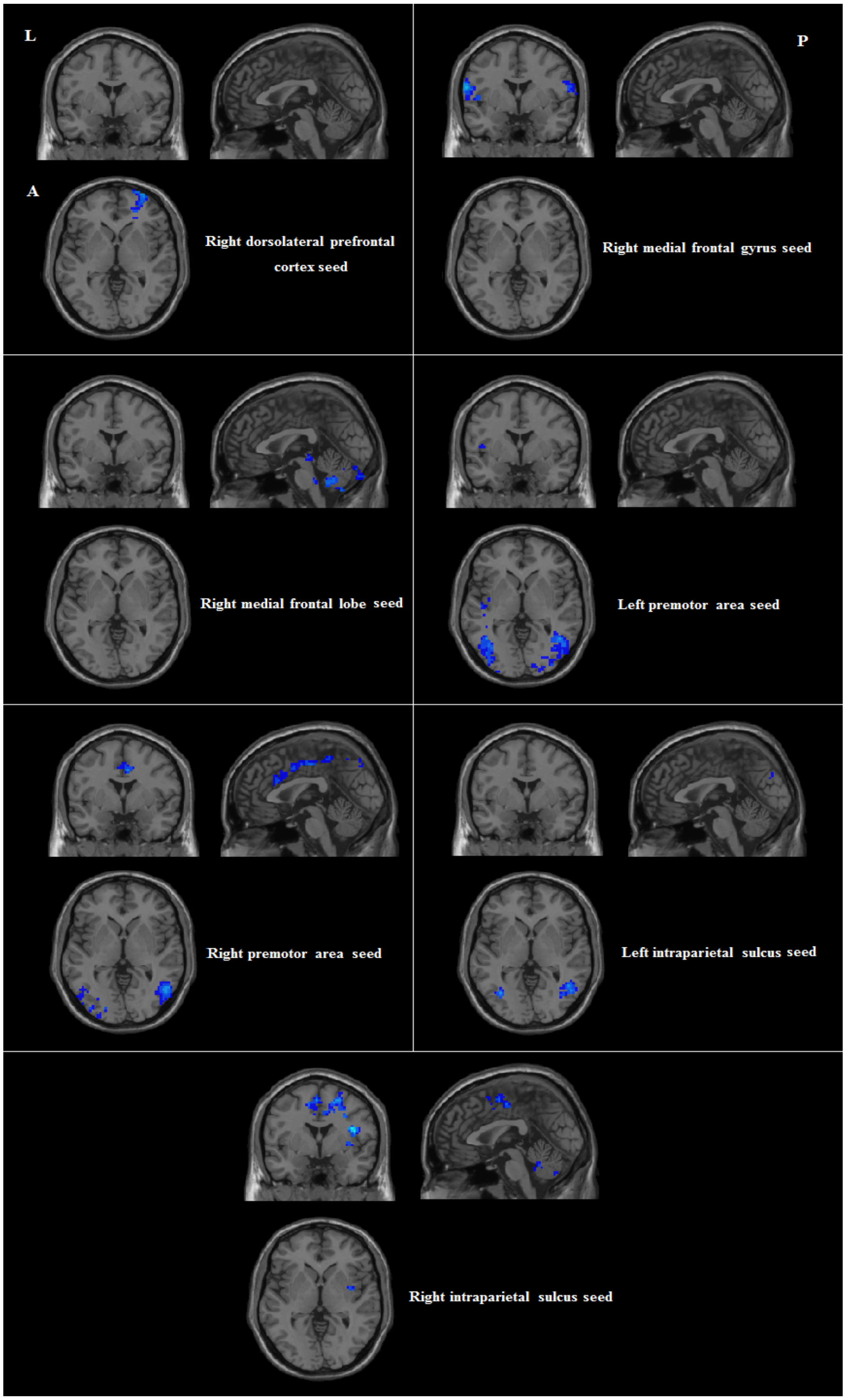

Supplementary Figure S1. Differences in negative RSFC between participants with and without musical training (AlphaSim corrected $p < 0.05$, cluster size > 212). L: left; A: anterior; P: posterior.

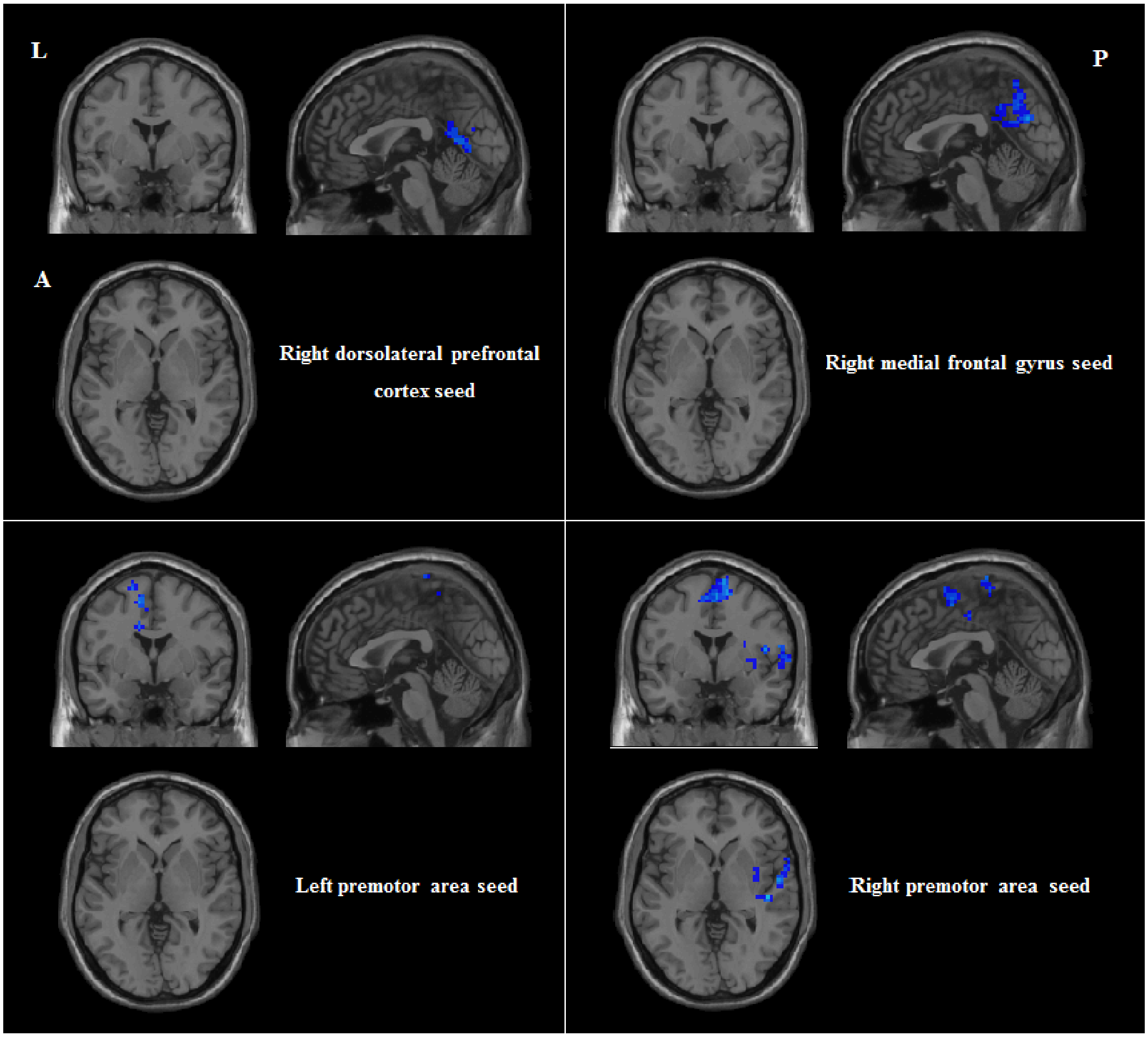

Supplementary Figure S2. Negative correlations between RSFC and PI for participants with musical training (AlphaSim corrected $p < 0.05$, cluster size > 212). L: left; A: anterior; P: posterior.

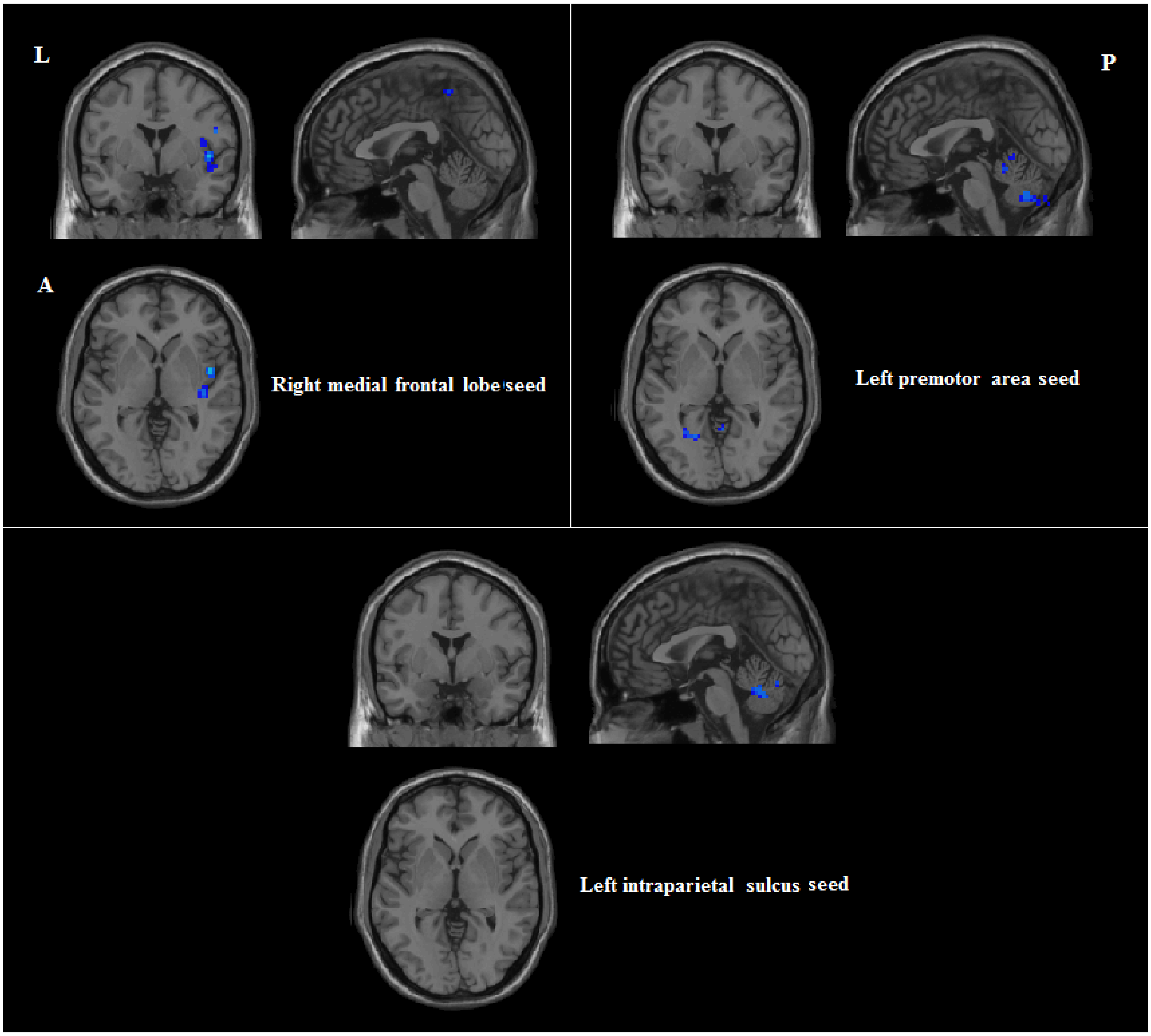

Supplementary Figure S3. Negative correlations between RSFC and PI for participants without musical training (AlphaSim corrected $p < 0.05$, cluster size > 212). L: left; A: anterior; P: posterior.

Supplementary Table S1. Negative differences in RSFC between participants with and without musical training.

ROI seeds	Cluster location	BA	Peak (MNI)			Cluster size	*t*
			x	*y*	*z*		
Right dorsolateral prefrontal cortex	Right orbitofrontal area	11	21	69	-6	467	-4.08
Right medial frontal gyrus	Right middle occipital gyrus	19	27	-75	6	109	-4.32
	Left precentral gyrus	6	39	-15	45	256	-3.30
Right medial frontal lobe	Left cerebellum		-42	-69	-45	1209	-4.21
Left premotor area	Right postcentral gyrus	3	39	-33	51	259	-3.59
	Right inferior temporal gyrus	37	48	-66	-9	935	-4.81
	Left inferior temporal gyrus	37	-42	-63	-9	961	-4.34
Right premotor area	Right middle temporal gyrus	37	51	-63	-9	431	-4.31
	Right superior occipital gyrus	7	21	-66	36	1598	-4.85
	Left cerebellum		-33	-54	-54	318	-4.22
	Left inferior occipital gyrus	37	-51	-69	-6	271	-3.50
Left intraparietal sulcus	Right inferior temporal gyrus	37	45	-63	-6	271	-3.42
	Left middle occipital gyrus	19	-21	-72	18	363	-3.28
Right intraparietal sulcus	Right postcentral gyrus	7	33	-48	72	227	-3.65
	Right retrosubicular area	48	39	0	21	407	-3.78
	Right vermis		6	-48	-30	412	-3.60

Note: AlphaSim corrected $p < 0.05$, cluster size > 212.

Supplementary Table S2. Negative correlations between RSFC and PI for the two groups of participants.

ROI seeds	Cluster location	BA	Peak (MNI)			Cluster size	*r*
			x	*y*	*z*		
**Participants with musical training**							
Right dorsolateral prefrontal cortex	Right cuneus	18	21	-72	24	277	-0.44
Right medial frontal gyrus	Left middle cingulum	23	-12	-45	33	372	-0.50
Left premotor area	Left paracentral lobule	4	-9	-36	78	310	-0.43
Right premotor area	Right retrosubicular area	48	48	-3	18	339	-0.46
	Right postcentral gyrus	4	15	-36	75	865	-0.49
**Participants without musical training**							
Right medial frontal lobe	Right cuneus	7	18	-63	39	598	-0.21
	Right superior temporal gyrus	48	39	-18	9	271	-0.26
	Left inferior parietal lobule	40	-42	-42	54	309	-0.22
Left premotor area	Right cerebellum		9	-66	-45	317	-0.25
	Left cerebellum		-24	-57	-18	266	-0.25
Left intraparietal sulcus	Left cerebellum		-6	-69	-15	249	-0.21

Note: AlphaSim corrected $p < 0.05$, cluster size > 212.

Supplementary Table S3. Differences in RSFC with whole-brain AAL ROIs between participants with and without musical training.

ROI seeds	Cluster location	BA	Peak (MNI)	Cluster	*t*

			x	*y*	*z*	**size**	
Right middle frontal gyrus	Right superior temporal gyrus	21	51	0	-24	232	3.86
	Right superior temporal pole	38	48	21	-21	270	3.15
	Right inferior parietal lobule	40	36	-45	40	215	3.64
Right precentral gyrus	Right inferior temporal gyrus	20	51	0	-39	246	3.59
	Left middle temporal gyrus	21	-66	-27	-12	319	4.55
Right insula	Right precentral gyrus	8	33	15	12	350	-3.88
	Left superior frontal gyrus	11	-18	33	45	374	3.58
Right parahippcompal gyrus	Right cerebellum_4_5		15	-48	-15	251	-3.11
	Left fusiform	36	-30	-33	-15	255	-4.95
Right thalamus	Right posterior cingulum		9	-39	18	344	-4.77
	Right cuneus	31	15	-69	27	256	-3.85
Right inferior parietal lobule	Left inferior frontal triangular	45	-42	36	-15	317	3.09
	Right insula	48	39	-9	15	221	4.62
Left supsrior medial frontal gyrus	Left rectus	11	-3	51	-21	334	-4.22
Left middle frontal gyrus	Left superior orbitofrontal gyrus	11	-21	48	-9	221	4.39
Left precentral gyrus	Left cerebellum_crus_1		-15	-78	-27	314	4.78
	Left middle temporal gyrus	37	-36	-66	12	373	-4.22
	Left anterior cingulum	10	-12	33	-3	215	4.00
Left inferior parietal lobule	Left middle frontal gyrus	45	-24	42	20	318	-3.73

Note: AAL: Automated Anatomical Labeling. AlphaSim corrected $p < 0.05$, cluster size > 212.

Discussion of *Supplementary Table S3*

First, the AAL ROI results confirmed the original ROI analyses in terms of the importance of the middle frontal gyrus, precentral gyrus, and inferior parietal lobule as seed regions. As discussed in the main text, these regions are responsible for emotion processing, coupling the moment-to-moment perception of tonal space with cognitive and motoric associations, and spatial cognition, respectively. In addition, AAL ROI analysis showed significant results for seed regions such as the insula gyrus, which is responsible for motor control (Mutschler et al., 2007) and emotion (Craig, 2002), and the thalamus ,which processes sensory information (Alitto et al., 2003). Taken together, these results may reflect the role of music training in improving more

cognitive and neural functions, including vision, auditory, spatial cognition, movement perception, and emotional perception, as found in previous studies (e.g., Zhou, 2004; Schmithorst, 2005; Moreno et al., 2011).

References of *Supplementary Table S3*

Alitto, H., and Usrey, W.M. (2003). Corticothalamic feedback and sensory processing. *Curr Opin Neurobiol.* 13, 440–445. doi: 10.1016/S0959-4388(03)00096-5

Craig, A. D. (2002). A new view of pain as a homeostatic emotion. *Trends Neurosci.* 26, 303–307. doi:10.1016/S0166-2236(03)00123-1

Moreno, S., Bialystok, E., Barac, R., Schellenberg, E.G., Cepeda, N.J., and Chau, T. (2011). Short-term music training enhances verbal intelligence and executive function. *Psychological Science* 22, 1425–1433. doi: 10.1177/0956797611416999

Schmithorst, V.J. (2005). Separate cortical networks involved in music perception: preliminary functional MRI evidence for modularity of music processing. *Neuroimage* 25, 444–451. doi: 10.1016/j.neuroimage.2004.12.006

Zhou, H.H. (2004). *The music and the words of related expressions (in Chinese)*. Beijing: College of Center Music Press.

Supplementary Table S4. Correlations between RSFC with whole-brain AAL ROIs and PI for the two groups of participants.

ROI seeds	Cluster location	BA	Peak (MNI)			Cluster size	*r*
			x	*y*	*z*		
**Participants with musical training**							
Right precentral gyrus	Left cuneus	18	-20	-69	27	433	-0.41
	Left middle cingulum	23	-12	-45	33	332	-0.46
	Left cerebellum_8		36	-66	-51	220	0.51
Right middle frontal gyrus	Left precentral lobule	6	-12	-30	72	236	0.57
	Right precuneus	7	3	-66	51	390	-0.44
Right superior frontal gyrus	Left middle temporal gyrus	20	-54	-21	-15	313	0.58
	Left superior orbitofrontal cortex	11	-18	66	-9	1819	0.50
	Left insula	48	-36	-3	0	381	-0.46
	Left precentral gyrus	6	-18	-12	69	286	-0.55
	Right inferior temporal gyrus	20	57	6	-36	371	0.47
	Left cerebellum_6		-15	-81	-21	213	0.45
Right angular gyrus	Right middle frontal gyrus	10	54	18	45	265	0.51
	Left middle orbitofrontal gyrus	47	-48	42	-15	228	0.37
	Right insula	13	33	15	12	777	-0.47
	Right posterior cingulate cortex	23	0	-12	48	286	-0.54
	Right precentral gyrus	3	15	-30	57	219	-0.60
Right superior temporal gyrus	Right superior orbitofrontal gyrus	8	51	33	-9	227	0.33
	Right middle frontal gyrus	11	27	57	0	216	0.42
Right insula	Right superior medial frontal cortex	10	12	48	6	300	0.48
Right inferior parietal lobule	Right superior medial frontal cortex	32	9	36	42	272	0.47
Left precentral gyrus	Left middle orbitofrontal cortex	11	-36	60	-9	263	0.54
	Right superior medial frontal cortex	32	10	40	31	269	0.47
	Left superior temporal gyrus	42	-66	-12	6	413	-0.50
Left medial orbitofrontal cortex	Left inferior parietal lobule	40	-39	-33	36	552	-0.55
	Right superior parietal lobule	7	39	-33	39	214	-0.50
	Left cerebellum_8		-12	-63	-45	229	-0.48
Left superior frontal gyrus	Left insula	48	-36	-6	3	376	-0.45
Left supramarginal gyrus	Left inferior frontal triangularis	23	-4	-29	30	239	-0.44
	Left inferior parietal lobule	40	-36	-45	39	215	-0.64
	Right cerebellum_crus_2		36	-72	-45	246	0.55
Left superior parietal lobule	Left cerebellum_crus_1		-39	-45	-33	244	0.51

	Right inferior temporal gyrus	20	60	-24	-30	426	0.43
	Right inferior frontal gyrus	44	33	15	36	320	0.57
Left superior temporal gyrus	Left superior orbitofrontal cortex	47	-51	33	-12	278	0.39
Left precuneus	Right middle temporal gyrus	37	45	-63	6	351	-0.55
	Left inferior temporal gyrus	20	-63	-21	-27	272	0.54
	Left insula	48	-30	9	12	336	-0.43
	Right insula	13	33	9	12	307	-0.44
**Participants without musical training**							
Right middle frontal gyrus	Left putamen	48	-21	15	-9	287	0.23
	Right supplementary motor area	6	12	-3	60	312	-0.25
Right inferior frontal triangularis	Right middle cingulum	23	3	-19	32	246	-0.23
Right superior medial frontal gyrus	Right precuneus	7	9	-60	60	417	0.25
	Left superior temporal gyrus	22	-66	-6	3	375	-0.31
Right inferior temporal gyrus	Right superior parietal lobule	40	45	-45	60	248	0.25
	Left caudate	25	-9	12	-12	238	-0.29
Right superior temporal gyrus	Left cerebellum_6		-30	-39	-30	603	-0.28
	Right inferior frontal triangularis	44	57	12	18	-246	-0.32
	Left supplementary motor area	6	-6	27	63	618	0.28
	Right supramarginal gyrus	48	48	-33	27	700	-0.35
Right fusiform	Right precentral gyrus	8	51	12	42	300	0.25
Right inferior parietal lobule	Right inferior temporal gyrus	21	45	9	-45	274	0.27
	Right putamen	48	21	-2	3	253	-0.25
	Right superior temporal gyrus	42	60	-27	18	452	-0.26
Right superior parietal lobule	Right superior temporal pole	22	69	-18	0	216	0.24
Left parahippocampal	Left calcarine	18	-6	-90	-12	654	-0.31
	Right precuneus	7	9	-60	42	517	0.25
	Right superior frontal gyrus	8	18	30	42	220	0.26
	Left middle cingulum	24	-12	-18	48	506	-0.25
	Right precentral gyrus	6	21	-24	69	272	-0.25
Left precentral gyrus	Right superior orbifrontal gyrus	6	63	-9	-24	261	0.22
Left middle frontal gyrus	Left parahippocampal	30	-27	-21	-27	222	0.28
	Left calcarine	30	-12	-54	12	236	0.22
Left middle temporal gyrus	Right oper rolandic	44	48	0	6	230	0.26
Left inferior parietal lobule	Right superior parietal lobule	7	21	-69	51	258	-0.28
	Left inusla	47	-33	20	-7	239	0.27
Right inferior parietal lobule	Right superior temporal gyrus	22	48	-33	21	847	-0.30

	Left caudate	11	-3	12	-3	443	0.26
	Left superior temporal gyrus	22	-51	3	-6	236	-0.29
	Left middle frontal gyrus	8	-24	24	51	278	0.28

Note: AAL: Automated Anatomical Labeling. AlphaSim corrected $p < 0.05$, cluster size > 212.

Discussion of *Supplementary Table S4*

In participants with music training, the AAL ROI results confirmed the original ROI analyses in terms of the importance of the precentral gyrus as a seed region. In addition, AAL ROI analysis showed significant results for the following seed regions. First, consistent with the results from the comparison of participants with and without music training (see Table S3), the middle frontal gyrus and the insula seemed to play an important role in PI perception. Second, additional seed regions also showed significant results, including the superior temporal gyrus, which important for sound perception and categorization, as well as speech processing (Loui et al., 2012), and the angular gyrus which is important for musical emotion (Schmithorst, 2005) as well as language and spatial cognition in musicians (Koelsch et al., 2007).

In participants without musical training, the AAL ROI results also confirmed the original ROI analyses in terms of the importance of the precentral gyrus as a seed region. In addition, AAL ROI analysis showed significant results for seed regions such as the inferior temporal gyrus which is responsible for vision and auditory processing (Hasler et al., 2007); the parahippocampal gyrus which is involved in memory encoding, retrieval, and emotion processing (Blood and Zatorre, 2001); and the superior medial frontal gyrus which is involved in executive functions (Talati and Hirsch, 2005).

References of *Supplementary Table S4*

Blood, A.J., and Zatorre, R.J. (2001). Intensely pleasurable responses to music correlate with activity in brain regions implicated in reward and emotion. *Proc. Natl. Acad. Sci. U.S.A.* 98, 11818–11823. doi: 10.1073/pnas.191355898

Hasler, G., Fromm, S., Alvarez, R.P., Luckenbaugh, D.A., Drevets, W.C., and Grillon, C. (2007). Cerebral blood flow in immediate and sustained anxiety. *J Neurosci.* 27, 6313–6319. doi:10.1523/JNEUROSCI.5369-06.2007

Koelsch, S., Fritz, T., Cramon, D., Muller, K., and Friederici, A. (2007). Prefrontal involvement in imitation learning of hand actions: effects of practice and expertise. *Neuroimage* 37, 1371–1381. doi:10.1016/j.neuroimage.2007.07.005

Loui, P., Zamm, A., and Schlaug, G. (2012). Enhanced functional networks in absolute pitch. *Neuroimage,* 63, 632–640. doi: 10.1016/j.neuroimage.2012.07.030

Schmithorst, V.J. (2005). Separate cortical networks involved in music perception: preliminary functional MRI evidence for modularity of music processing. *Neuroimage*, 25, 444–451. doi: 10.1016/j.neuroimage.2004.12.006

Talati, A., and Hirsch, J. (2005). Functional specialization within the medial frontal gyrus for perceptual go/no-go decisions based on “what,” “when,” and “where” related information: an fMRI study. *J Cogn Neurosci.* 17, 981–993. doi: 10.1162/0898929054475226
